# Embedded Machine Learning Using a Multi-Thread Algorithm on a Raspberry Pi Platform to Improve Prosthetic Hand Performance

**DOI:** 10.3390/mi13020191

**Published:** 2022-01-26

**Authors:** Triwiyanto Triwiyanto, Wahyu Caesarendra, Mauridhi Hery Purnomo, Maciej Sułowicz, I Dewa Gede Hari Wisana, Dyah Titisari, Lamidi Lamidi, Rismayani Rismayani

**Affiliations:** 1 Department of Medical Electronics Technology, Poltekkes Kemenkes Surabaya, Surabaya 60282, Indonesia; dewa@poltekkesdepkes-sby.ac.id (I.D.G.H.W.); ti2sari@poltekkesdepkes-sby.ac.id (D.T.); lamidi@poltekkesdepkes-sby.ac.id (L.L.); 2 Manufacturing Systems Engineering, Faculty of Integrated Technologies, Universiti Brunei Darussalam, Gadong BE1410, Brunei; 3 Department of Computer Engineering, Institute of Sepuluh Nopember, Surabaya 60111, Indonesia; hery@ee.its.ac.id; 4 Department of Electrical Engineering, Cracow University of Technology, 31-155 Cracow, Poland; msulowicz@pk.edu.pl; 5 Department of Software Engineering, Dipa Makassar University, Makassar 90245, Indonesia; rismayani@undipa.ac.id

**Keywords:** multi-thread, embedded system, Raspberry Pi, EMG, machine learning, time-domain feature, prosthetic hand

## Abstract

High accuracy and a real-time system are priorities in the development of a prosthetic hand. This study aimed to develop and evaluate a real-time embedded time-domain feature extraction and machine learning on a system on chip (SoC) Raspberry platform using a multi-thread algorithm to operate a prosthetic hand device. The contribution of this study is that the implementation of the multi-thread in the pattern recognition improves the accuracy and decreases the computation time in the SoC. In this study, ten healthy volunteers were involved. The EMG signal was collected by using two dry electrodes placed on the wrist flexor and wrist extensor muscles. To reduce the complexity, four time-domain features were applied to extract the EMG signal. Furthermore, these features were used as the input of the machine learning. The machine learning evaluated in this study were *k*-nearest neighbor (*k*-NN), Naive Bayes (NB), decision tree (DT), and support vector machine (SVM). In the SoC implementation, the data acquisition, feature extraction, machine learning, and motor control process were implemented using a multi-thread algorithm. After the evaluation, the result showed that the pairing of the MAV feature and machine learning DT resulted in higher accuracy among other combinations (98.41%) with a computation time of ~1 ms. The implementation of the multi-thread algorithm in the pattern recognition system resulted in significant impact on the time processing.

## 1. Introduction

Prosthetic hands are needed for people who have amputations on the hand (amputee) due to work accidents, congenital defects, and certain diseases. The need for prosthetic hands is still high, especially in developing countries and countries with war conflict [1,2]. More importantly, prosthetic hands are not only used cosmetically but also have a functional role to help the amputee with daily life activities [3,4]. The development of prosthetic hands has been carried out by previous studies both in the laboratory and at industrial levels. In the laboratory stage, previous studies developed a prosthetic hand that had a greater emphasis on control systems including the sensor system and pattern recognition. At the industrial level (i-Limb [5], Bebionic [6], and Vincent [7]), researchers emphasized research on battery management systems as well as portability and functionality. Combining the two demands remains a challenge for researchers, namely, how to develop a system that can recognize hand movement patterns based on EMG signals by applying a machine learning algorithm to the system on chip (SoC) so that functionality and portability can be achieved on the prosthetic hand device.

Several previous studies have developed prosthetic hands using bioelectric signals such as electroencephalography (EEG), electrooculography (EOG), electromyography (EMG), and a vision system [8,9,10,11]. Two methods are commonly used to control prosthetic hands, namely, threshold- [12,13] and pattern recognition-based [14,15]. The threshold-based method cannot recognize the complexity of the EMG signal when it is used to identify more than two movements. Therefore, the development of a controlled prosthetic hand through the recognition of EMG signal patterns that include feature extraction and machine learning-based classifiers is a major concern for researchers [16,17]. Several previous researchers have succeeded in developing prosthetic hands with good accuracy (>90%) but most of these researchers used more than two electrodes [18,19,20,21,22]. Chen et al. developed a prosthetic hand using the SVM as a classifier and obtained an accuracy of 90% [19]. However, Chen et al. still used personal computer equipment to implement the feature extraction and classifier process. Another prosthetic hand development was carried out by Lee at al. The researchers used an artificial neural network (ANN) classifier to detect six different hand movement patterns using eight EMG channels and obtained an accuracy value of 93.20% [21]. In this study, the researchers also used a personal computer to implement the feature extraction and classifier processes. Tavakoli et al. developed a prosthetic hand using a single-channel EMG to recognize four movement patterns (open, close, change, and lock). However, the average accuracy obtained was 90.1%. In addition, this research was developed using a feature extraction and classification process using a computer system [23]. A portable system is a demand in the development of prosthetic hands; therefore, several researchers have developed embedded machine learning for the process of recognizing EMG signal patterns on prosthetic hand devices. Benatti developed a prosthetic hand by implementing the SVM classifier on the microcontroller unit (MCU) with an accuracy of 90% using eight channels of the EMG signal [20]. Additionally, Pancholi et al. developed a prosthetic hand by applying the LDA classifier to the DSP MCU with an average accuracy of 94.14%. Gaetani et. al. developed a prosthetic hand with open-source Adam by applying the ANN classifier to the SoC of a Raspberry Pi 3B+ board [24]. However, this research did not carry out a detailed investigation into the features and machine learning used so there was no information obtained related to the performance of the system that was developed. Another researcher developed a prosthetic arm with a SoC-based architecture using the Xilinx ZYNQ FPGA platform. This study resulted in an average accuracy of 92% for three movements (relaxing, clasping, and stretching) [25]. However, the use of FPGA as SoCs for the development of embedded features and classifiers has limitations in terms of programmable logic resources. Souza et al. developed a real-time system classifier using eight myo-arm electrode channels and a multi-layer perceptron that was implemented on a Raspberry Pi platform [26]. The average accuracy to discriminate 11 motion patterns was 96.11%.

Based on the literature described, it is clear that further development of prosthetic hands based on pattern recognition using a machine classifier is recommended. The development of embedded machine learning on the SoC provides added value, especially in portability and effectiveness, when it is implemented on prosthetic hands. To the best of our knowledge, several studies have developed a prosthetic hand based on a real-time system classifier using an embedded system to control the motors connected to the finger. However, the classifier system in the prosthetic hand that controls the finger and wrist movements has not been widely studied. Additionally, high accuracy and the ability of the system to process in real-time are very important. Therefore, this study aims to develop and evaluate an embedded time-domain feature and machine learning on a system on chip (SoC) based on a Raspberry platform using a multi-thread algorithm to control the prosthetic hand device to recognize four motions (grasp, relax (open), wrist flexion, and wrist extension). Raspberry Pi is a SoC that has a Raspbian operating system with the ability to run multiple tasks concurrently or in parallel. The process of running programs in parallel can be implemented using the threading library in Python programming. In this study, the data acquisition, feature extraction, classifier, and driving motor processes are implemented using a multi-threading algorithm so that the system runs in real-time and produces a good system performance.

## 2. Materials and Methods

### 2.1. Minimum System

**Figure 1:**
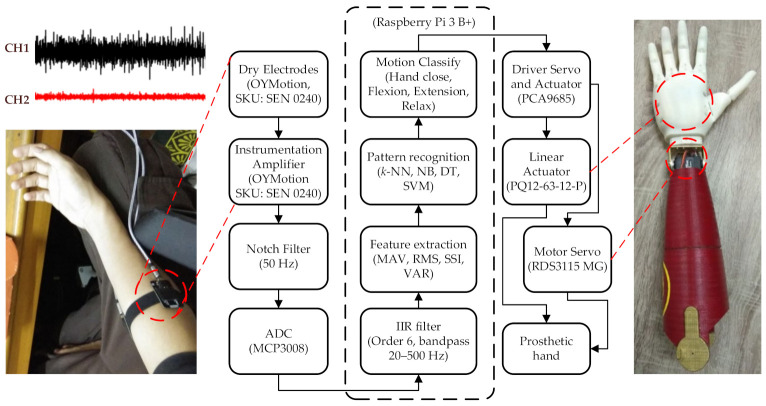
The block diagram system for a prosthetic hand.

In this study, embedded machine learning was applied to a SoC Raspberry Pi 3 B+ system (Figure 1). To collect the EMG signal, two dry electrodes (OYMotion SEN 0240, Shanghai, China) were placed on the wrist flexor and wrist extensor muscles. The dry electrode had a built-in instrumentation amplifier. These two muscles are the most involved in hand grasp, relax (open), wrist flexion, and wrist extension movements [27]. A 50 Hz notch filter was applied to the EMG signal to reduce the noise added by the line power. The Raspberry Pi family does not have an analog to digital (AD) feature. Therefore, in this data acquisition process, the EMG signal was recorded using an external AD (MCP 3008, Microchip, Chandler, AZ, USA).

In this research, a Broadcom BCM2835 system on chip (SoC) was used with an ARM1176JZF-S processor, a 700 MHz clock, a VideoCore IV GPU, and 256 MB RAM. The digital filter, feature extraction, and machine learning processes were carried out in a Raspberry Pi system. Additionally, the digital data from the A/D MCP3008 (10 bit ADC) were then filtered using a digital bandpass filter based on an infinite impulse response (IIR) with a cut-off frequency of 20–500 Hz, order 6. In the investigation stage, several time-domain features and machine learning methods were evaluated to find the best performance. In this study, the time-domain features used were mean absolute value (MAV), root mean square (RMS), simple square integral (SSI), and variance (VAR) [28]. The machine learning methods that were evaluated in this research were *k*-nearest neighbor (*k*-NN), Naive Bayes (NB), decision tree (DT), and support vector machine (SVM). In this case, the classifier built using machine learning recognized movement patterns through the EMG feature. Furthermore, the classifier instructed several servo motors to perform movements according to the movement pattern being trained. When the system detected an open or grasp movement, five linear actuators (PQ12P, Actuonix, Victoria, BC, Canada) in the palm were activated simultaneously so that it produced a grasp or open hand movement mechanism. Additionally, when the system detect wrist flexion or an extension movement, the servo motor (RDS RDS3115 MG, Robot Digital Servo, India) on the prosthetic arm responded according to the motion received (wrist flexion or wrist extension). The RDS3115 servo motor had a weight of 100 g and a torque of 15 kg so it was light and strong enough to hold and perform the motion for a hand wrist flexion and extension.

The prosthetic hand was printed using a 3D printer machine (Creality CR-6, Creality, Shenzhen, China). In this study, the prosthetic hand consisted of two parts; the first part was the 3D printing for the upper arm, made of polylactic acid (PLA, 30% infill) and the second part was the 3D printing for the hands and fingers, made of a flexible filament material (TPU, 95% infill). To connect the upper arm and the hand, a servo motor was permanently attached to the wrist upper arm and held the hand. Moreover, in the hand part, there were five linear actuators placed in the hand palm, each of which moved a prosthetic finger.

### 2.2. Proposed Method

The implementation of data acquisition, feature extraction, classification, and motor control processes on the prosthetic hand control was carried out in parallel on the SoC Raspberry Pi machine to ensure that the system could run in real-time. Several parallel tasks can be implemented on a Raspberry Pi SoC by running several threads simultaneously, known as multi-threading. A Python programming application was supported by a threading library so that it made the implementation easier (https://github.com/triwiyanto/Prosthetic-Pi (accessed on 10 December 2021)).

**Figure 2:**
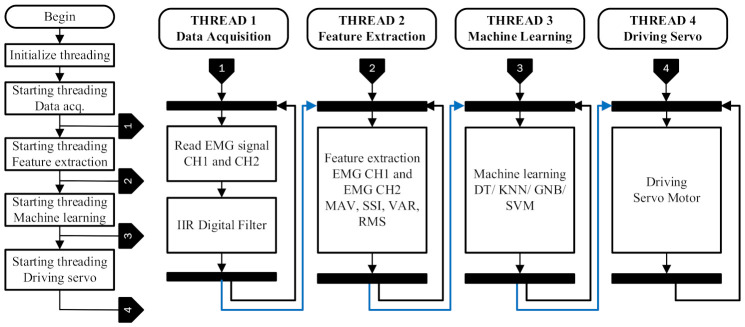
Embedded feature extraction and machine learning process on a SoC using a multi-thread algorithm.

The implementation of multi-threading on the prosthetic hands was implemented by creating four threads, namely, thread data acquisition, feature extraction, machine learning, and motor control. For each variable output from a thread to be recognized by other threads, all related variables had to be declared globally in the Python programming. The main program (Figure 2) was responsible for initializing and starting each thread. In this implementation, the threading data acquisition was the first process. The threading data acquisition worked continuously and recorded two channels of the EMG signal with a sampling frequency of 1000 Hz. This sampling frequency rate was chosen according to the Nyquist requirement [29]. Additionally, the EMG signal was segmented with a window length of 100 ms [30,31]. Furthermore, in this thread, the EMG signal was filtered using an IIR digital filter (order 6, bandpass filter 20–500 Hz). A digital filter using an IIR basis was implemented using Equation (1) [32,33].

(1) $$EMG_{f}^{CH}\left[k\right]=b_{0}EMG\left[k\right]+b_{1}EMG\left[k-1\right]+b_{2}EMG\left[k-2\right]+..+b_{M}EMG\left(k-M\right)\;-a_{1}EMG\left[k-1\right]-a_{2}EMG\left[k-2\right]-..-a_{N}EMG\left[k-N\right]$$

where $EMG_{f}^{CH}[k]$ indicates the filtered EMG signal for channel CH and EMG[k] shows the *k* EMG signal (raw data). Additionally, *b_0_*, *b_1_*, *b_2_*,…, *b_M_* and *a_1_*, *a_2_*,…, a*_N_* indicates the bandpass filter coefficients.

Threading feature extraction was also begun and carried out the feature extraction process for two filtered EMG signals. In this thread, two EMG signal channels were extracted using the RMS, VAR, MAV, and SSI features, which are described in Equations (2)–(5), respectively [28,34,35,36]. The result of the feature extraction was then used by other threads because it had been globally defined. This threading feature extraction ran continuously as long as the system was not stopped.

(2) $$RMS=\sqrt{\frac{1}{N}\sum_{i=1}^{N}EMG_{f}^{CH}{\left[k\right]}_{}^{2}}$$

(3) $$VAR=\frac{1}{N-1}\sum_{i=1}^{N}EMG_{f}^{CH}{\left[k\right]}_{}^{2}$$

(4) $$MAV=\frac{1}{N}\sum_{i=1}^{N}\left|EMG_{f}^{CH}[k]\right|$$

(5) $$SSI=\sum_{i=1}^{N}EMG_{f}^{CH}{\left[k\right]}^{2}$$

where $EMG_{f}^{CH}\left[k\right]$ indicates the *k* filtered EMG signal, CH is the EMG channel, *f* is feature, and *N* is the number of collected data.

Threading machine learning was pre-started and ran continuously. When the thread did not obtain the latest data, machine learning retrieved the data from the pre-defined or the last updated data. At the training stage, all machine learning—including *k*-NN, NB, SVM, and DT—were used and evaluated but in the implementation stage, only feature and machine learning were selected based on the one that had the highest average accuracy. In this study, *k*-NN was also applied as a classifier machine because it had a simple algorithm and performed a classification process based on the similarity of the measure distance. Generally, the Minkowski distance was used as the default distance in the Python programming. It measured the distance between the classes, as shown in Equation (6) [36]:

(6) $$d(x,y)={\left(\sum_{i=1}^{n}|x_{i}-y_{i}{|}^{c}\right)}^{\frac{1}{c}}$$

where *d*(*x*,*y*) show the distance, *x* and *y* are the features, and *c* is a constant. From the equation, when c was equal to 1 then the formula was the same as the Manhattan distance. When *c* was equal to 2, the formula was the same as the Euclidean distance [37]. Furthermore, in the programming implementation of *k*-NN, the *k* was chosen from 5. Naive Bayes (NB) machine learning was chosen because it has iterative parameters that are not complex and can be applied to large data. Posterior probability using the Bayes theorem is shown in Equation (6) where feature *X* is represented by Equations (7) and (8) [38]. In the Python programming, we used the “prior = none” and exponential constant of 1 × 10^−9^ parameters to configure the NB classifier.

(7) $$P(y|X)=\frac{P(X|y)P(y)}{P(X)}$$

(8) $$X=(x_{1},x_{2},x_{3},\ldots \ldots ,x_{n}).$$

Thus, based on Equations (7) and (8), the probability was obtained based on the number of features, as shown in Equation (9). Furthermore, the Gaussian Naïve Bayes used Equation (10) to determine the prediction.

(9) $$P(y|x_{1},x_{2},x_{3},\ldots \ldots ,x_{n})=\frac{P(x_{1}|y)P(x_{2}|y)\ldots P(x_{n}|y)P(y)}{P(x_{1})P(x_{2})\ldots P(x_{n})}$$

(10) $$y=\arg\max_{y}P(y)\prod_{i=1}^{n}P(x_{i}|y).$$

This research also implemented a support vector machine (SVM) to the Raspberry Pi SoC. A SVM has a light computation time and is easy to implement on single-processor machines as well as a small RAM capacity. The SVM ran the classification process by finding the right hyperplane that could maximize the margin between the classes with the optimization function shown in Equations (11) and (12) [39]. In the programming implementation, the radial basis function was used as the SVM kernel. The gamma parameter was set as “auto”.

(11) $$\max\sum_{i=1}^{n}b_{i}-\frac{1}{2}\sum_{i=1}^{n}\sum_{j=1}^{n}y_{i}b_{i}(x_{i}.x_{j})y_{j}b_{j}$$

(12) $$\mathrm{where}\;\sum_{i=1}^{n}b_{i}y_{i}=0\ldots and\ldots 0\leq b_{i}\leq \frac{1}{2ny}.$$

Another machine learning method that is often used in a SoC is the decision tree (DT). This classifier will break down the dataset into several subsets. The prediction on the DT classifier used the entropy calculation as shown in Equation (13) [17]. The entropy was used to measure the uncertainty of the dataset [40]. In the programming implementation, the maximum depth of the tree was chosen from three (max_dept = 3). This maximum depth served as a variable control for pre-pruning.

(13) $$E(s)=-\sum_{i=1}^{c}P(x_{i})\log_{2}P(x_{i})$$

where *P*(*x_i_*) shows the probability with the frequency that occurred frequently in class c.

The output of thread machine learning produced four codes that were then used by the motor control thread to move the prosthetic hand. These codes represented hand grasp, open, wrist flexion, and wrist extension movements. The threading driving servo was also started at the beginning and fetched data from pre-defined data before receiving updated data from thread machine learning. This thread ran continuously and controlled the prosthetic hand according to the updated data.

### 2.3. Data Collection

This study involved 10 volunteers who were in good health with an age range of 18 to 21 years old (20.45 ± 1.78 years) and a body weight of 55 to 65 kg (61.86 ± 3.67 kg). Each volunteer was given instructions to repeat each movement 10 times. The movements that had to be made were grasp, relax, wrist flexion, and wrist extension. Each transition between movements was given a delay of about 2–3 s; this was to prevent the occurrence of muscle fatigue. Two models of training testing machine learning were tested in this study: scheme 1, where all datasets originating from the 10 volunteers were collected into one and the data were separated by 80% for the training data and 20% for the testing data; and scheme 2, where the dataset was collected based on volunteers with 8 volunteers as the training data and 2 volunteers as the testing data. This research passed the ethical committee with ethical clearance number No. EA/078/KEPK-Poltekkes_Sby/V/2019.

The EMG signal was extracted using four time-domain feature extractions of MAV, RMS, SSI, and VAR. The output of the feature was used to train the machine learning. The four features were combined to produce fifteen feature combinations to train the machine learning. These 15 features combinations were MAV, RMS, SSI, VAR, MAV + RMS, MAV + SSI, MAV + VAR, RMS + SSI, RMS + VAR, SSI + VAR, MAV + RMS + SSI, MAV + RMS + VAR, MAV + SSI + VAR, RMS + SSI + VAR, and MAV + RMS + SSI + VAR. In addition to the feature evaluation, this study also evaluated four machine learning methods (*k*-NN, NB, DT, and SVM).

## 3. Results

**Figure 3:**
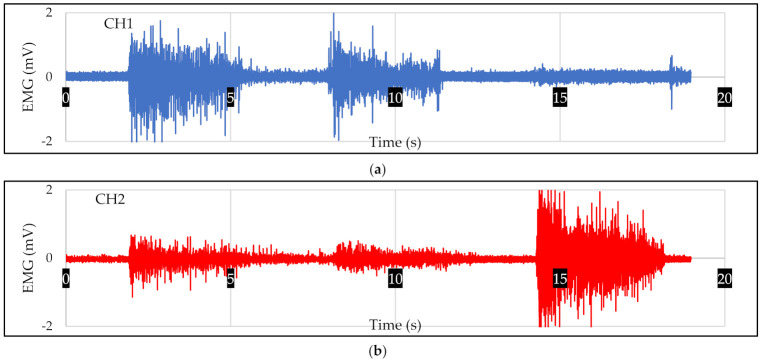
The recording of EMG signal from (**a**) wrist flexor and (**b**) wrist extensor. The EMG signal was recorded using frequency sampling of 1000 Hz. The EMG signal was recorded whilst the subject performed four hand motions. The sequences were grasp, wrist flexion, wrist extension, and relaxation.

In this study, the prosthetic hand functioned by using an EMG signal. An EMG signal has complex and random characteristics. Each different hand motion generated a different EMG pattern. Figure 3 shows the EMG pattern when the volunteer performed four hand motions (grasp, wrist flexion, wrist extension, and relax) sequentially. The EMG signal was collected from the wrist flexor (CH1) and wrist extensor (CH2).

The implementation of machine learning on the Raspberry Pi SoC using a multi-threading algorithm produced good accuracy (98.13%) and a fast computation time (~1 ms). Further details related to the research results are described in the following section.

### 3.1. Machine Learning Accuracy

**Figure 4:**
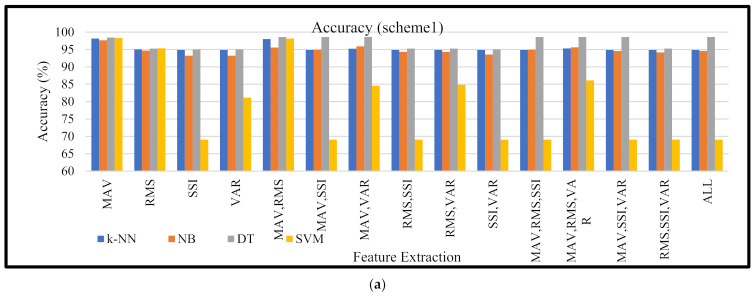

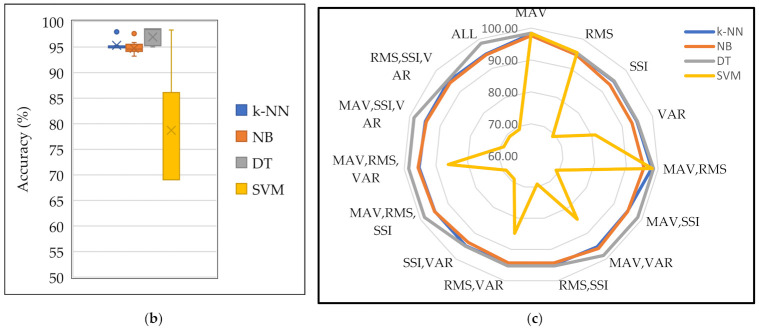
(**a**) Machine learning accuracy based on scheme 1 for different features and combinations, (**b**) the distribution of classifier accuracy based on all features used in this study (the cross sign indicates the mean value), and (**c**) the radar that shows the relation of the classifier accuracy and features used.

Figure 4a shows the accuracy of the machine learning based on single and combination features. These results used training data of 14,880 (80%) and 3731 (20%) of the total dataset based on scheme 1. The evaluation results showed that the MAV feature produced an average machine learning accuracy of 98.18%, which was higher than using other features or a combination of features. Among the machine learning evaluated, the decision tree produced the highest accuracy (98.42%) compared with the others. In the comparison among the classifier boxplots, Figure 4b shows the distribution of accuracy based on all features and their combination using a boxplot diagram. The cross sign indicates the mean accuracy value. The boxplot diagram shows that the *k*-NN, NB, and DT classifiers had a narrow box range (*k*-NN: 95.23–94.85%; NB: 94.16–95.55%; DT: 95.26–98.42%). On the other hand, the SVM boxplot diagram had a wide box range (69.04–86.12%). DT had a higher mean accuracy for all features and combinations than the other classifiers. Based on the radar graph (Figure 4c), this showed that DT produced a better accuracy compared with other classifiers. On the other hand, the accuracy of *k*-NN coincided with the accuracy of NB in almost all the features used.

**Figure 5:**
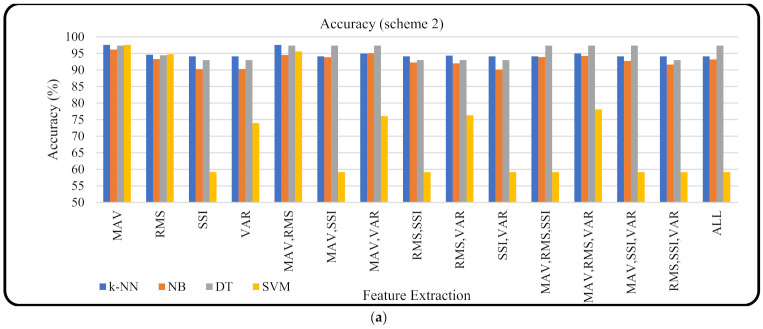

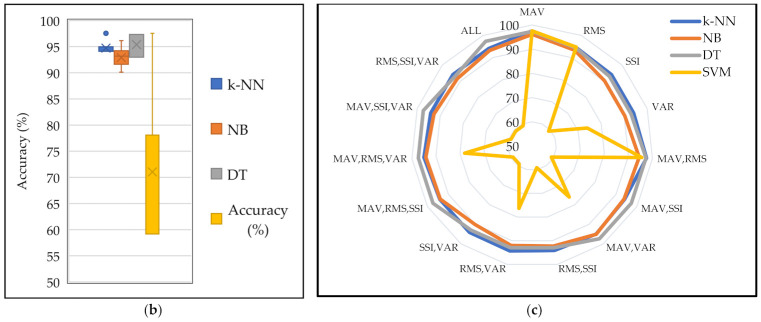
(**a**) Machine learning accuracy based on scheme 2 for different features and combinations, (**b**) the distribution of classifier accuracy based on all features used in this study (the cross sign indicates the mean value), and (**c**) the radar that shows the relation of the classifier accuracy and features used.

Figure 5 shows the accuracy of machine learning based on the features and their combinations that were applied for scheme 2. Eight volunteers were used as a training dataset (14,902 datasets) and two volunteers were used as a testing dataset (3715 datasets), (scheme 2); they had never been trained before. The results of the machine learning and features evaluation showed that the MAV features produced a higher accuracy than the others with an average accuracy of 97.15% (Figure 5a). As shown in the boxplot diagram (Figure 5b), *k*-NN, NB, and DT had a narrower width compared with SVM. Figure 5c shows that the accuracy of the *k*-NN, NB, and DT classifiers was almost the same for all the features used. However, the SVM classifier showed inconsistent accuracy in the features used.

In this study, two groups of accuracy from two different training schemes (schemes 1 and 2) were tested statistically using a *t*-test. In this test, only the accuracy data were taken from the DT classifier. The results showed that there was a significant difference in the accuracy produced by schemes 1 and 2 (*p*-value < 0.05). Furthermore, the best accuracy was produced by DT using scheme 1 (80% training and 20% testing).

### 3.2. Accuracy for Each Motion

**Figure 6:**
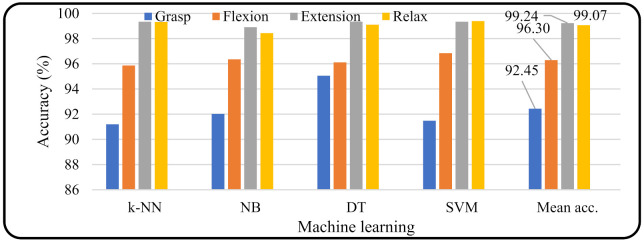
Machine learning accuracy based on type of motion.

In the evaluation of machine learning based on the features used, the MAV produced a higher accuracy than the other features and combinations. Therefore, in the subsequent evaluation, the MAV feature was used as the classifier input. Figure 6 shows the accuracy of machine learning (*k*-NN, NB, DT, and SVM) for every four motions (grasp, wrist flexion, wrist extension, and relax). The rightmost bar graph of Figure 6 shows the average accuracy value of all the machine learning methods for each motion. It appeared that motion extension produced a higher accuracy (99.24%) than the other motions (grasp = 92.45%, wrist flexion = 96.30%, and relax = 99.07%).

**Figure 7:**
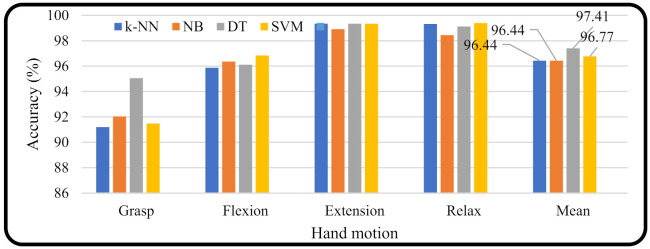
Accuracy for each motion based on machine learning applied.

Figure 7 shows a different perspective on the machine learning performance based on hand motion. The bar graph shows the results of the accuracy of each movement against the accuracy of machine learning. In the rightmost graph bar of Figure 7, it is shown that the machine learning decision tree (DT) produced a higher average accuracy for all hand motion types (97.41%) compared with the other machine learning methods (*k*-NN = 96.44, NB = 96.44, and SVM = 96.77).

**Figure 8:**
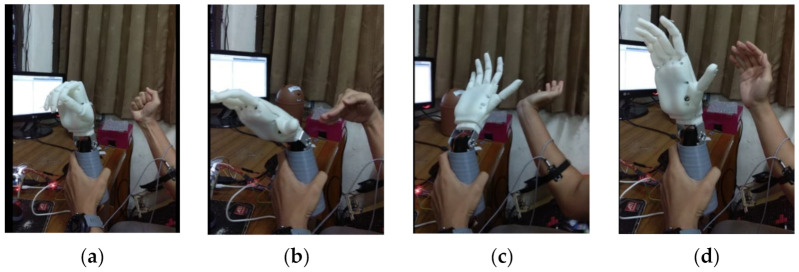
Implementation of embedded SoC on Raspberry Pi for hand motion classification: (a) grasp; (**b**) wrist flexion; (**c**) wrist extension; and (**d**) relax.

The overall results of the embedded machine learning on the Raspberry Pi machine can be seen in Figure 8. The test was carried out online by connecting the sensor to the Raspberry Pi. The Raspberry Pi system could also be connected to a computer device to detect movement activities.

### 3.3. Confusion Matrices

**Figure 9:**
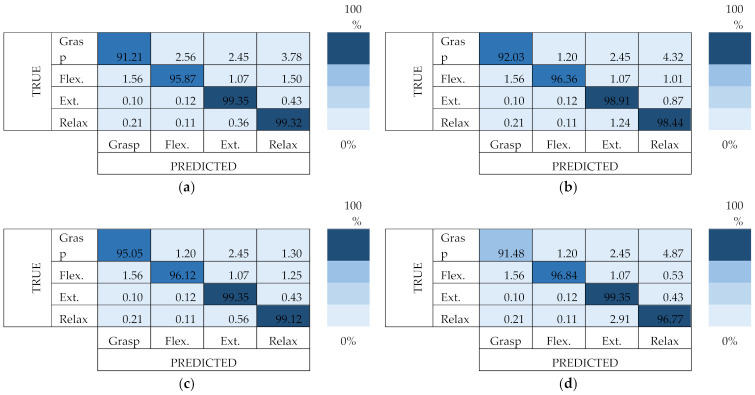
Confusion matrices for: (**a**) *k*-NN; (**b**) NB; (**c**) DT; and (**d**) SVM classifiers using the MAV feature.

A confusion matrix was performed based on the accuracy values generated by the classifier using the MAV feature, as shown in the previous section where it was demonstrated that the MAV feature resulted in the highest accuracy. The confusion matrix displayed the accuracy of each motion pattern (grasp, flexion, extension, and relax) as shown in Figure 9. Based on the three classifiers, the decision tree produced the highest average accuracy (97.41 ± 1.86%) for all motion patterns. The relax motion produced the highest accuracy for all classifiers (96.77–99.32%) followed by the extension, flexion, and grasp motion.

### 3.4. Computation Time

**Figure 10:**
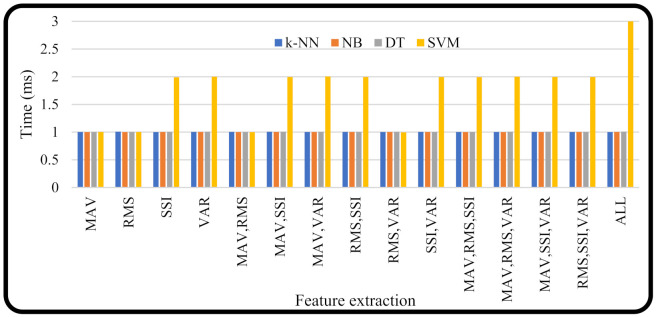
Computation time of a SoC on a Raspberry Pi.

Time computation is important for a system to know to discover whether the system can work in real-time or not. Testing was performed using the time library in Python. The computation time is the time required by the machine learning to classify the motion. The length of time was also tested with several combinations of existing features. Figure 10 shows that most of the average computational time required by *k*-NN, NB, and DT machine learning was about ~1 ms. The average computation time for SVM was 1.7989 ms.

## 4. Discussion

Based on the features and machine learning evaluation, MAV and decision tree (DT) produced a higher accuracy (98.41%) than the others. Therefore, these features and machine learning were implemented into the Raspberry SoC for the online prosthetic hand testing process. These results were also in line with the research conducted by Ma et al., who obtained high accuracy results when classifying four different movements by producing a maximum average accuracy value of 80% when using the MAV and RMS features [15].

**Figure 11:**
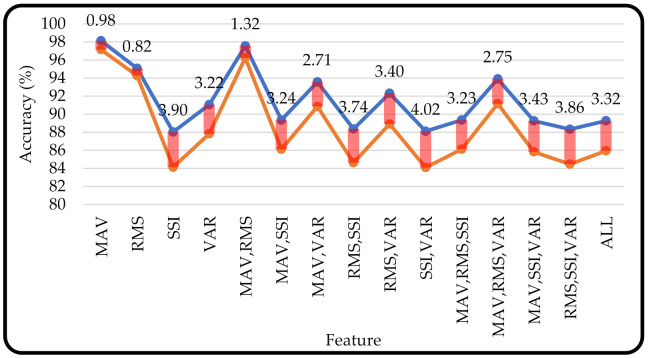
A comparison of training and testing scheme accuracy for different features and combinations. (Note: blue line = scheme 1 and red line = scheme 2).

Figure 11 shows that the accuracy of machine learning with the training and testing processes using scheme 1 (80% of the total dataset for training and 20% of the total dataset for testing) resulted in a higher accuracy than using scheme 2 (8 volunteers as training and 2 volunteers as testing). This showed that when the system was evaluated using scheme 2, the overall accuracy value decreased. This was reasonable because each volunteer had different EMG signal characteristics both in amplitude and frequency when the volunteer performed different hand motions. The change in the EMG characteristics was caused by the EMG signal that had stochastic and different characteristics for each person and time [41,42]. Thus, when the machine learning was tested using datasets from two volunteers who had never been trained before, the accuracy of machine learning decreased. Furthermore, machine learning accuracy with the MAV feature produced the smallest difference in accuracy (0.98%).

The overall performance of the proposed system was compared with the results of the studies that were undertaken, as described in Table 1. In the prosthetic hand implementation, several researchers used computers [19,21] and others used embedded systems [18,20] with the training process being carried out online or offline. Additionally, the testing process was all performed online. Previous studies used 4–8 EMG channels to recognize 6–9 motion patterns. After a comparison with other studies, it was shown that the proposed method produced accuracy that outperformed the other methods.

Figure 4 (scheme 1) and Figure 5 (scheme 2) show that a higher number of feature combinations could not increase the machine learning accuracy. This was proven when machine learning used a single MAV feature; the average accuracy of the four resulting machine learning methods was higher (accuracy = 98.13 ± 0.299%). Furthermore, when machine learning obtained an input from all feature combinations (MAV, RMS, SSI, and VAR), the average accuracy of machine learning was 89.27 ± 11.78%. This showed that each feature and its combination had a different suitability for the machine learning being used. This was not in line with the study conducted by Phinyomark et al., which showed that when the number of features was increased from 1 to 4, the accuracy increased slightly [35]. However, another study conducted by Phinyomark et al. showed that increasing the number of features was not always followed by an increase in accuracy; this depended on the classifier model used. In the study when an LDA classifier was applied there was a slight increase in accuracy but when the quadratic discriminant analysis classifier was used, the accuracy decreased after the number of features was increased from 2 to 3 and 4 [43].

Processing time is important when building a real-time system. Based on the results of the online testing of the Raspberry Pi SoC system, the average execution time was ~1 ms (for NB, *k*-NN, and DT); the average execution time required by machine learning SVM was longer (1.7989 ms). This execution time was aligned with the requirements required in building a real-time system, which is a maximum of 100 ms so that there is no delay between the input and output.

In this study, the implementation of feature extraction and machine learning on the Raspberry Pi SoC could run in real-time and online to control the prosthetic hand. However, a few limitations were not the attention of this study. In the data acquisition process, this study did not take into account the incidence of muscle fatigue caused by repeated measurement processes for each volunteer. As stated by previous researchers, the incidence of muscle fatigue appears when the volunteer performs repetitive movements [44]. Furthermore, when muscle fatigue occurs, it increases the amplitude and reduces the frequency of the EMG signal. Consequently, these EMG characteristic changes affect the resulting features as well as the machine learning accuracy. Regarding volunteers, this research only involved normal and healthy people; of course, this would be different if the system was used by amputees. This research was the evaluation phase of embedded machine learning on a Raspberry Pi SoC system. A larger number of volunteers for the training phase would certainly produce better results because machine learning recognizes various characteristics or patterns of additional EMG signals. The use of Raspberry Pi as an SoC system allowed wide opportunities.

In this study, the prosthetic hand used an open-source 3D printing design. (https://openbionicslabs.com/downloads, (accessed on 10 December 2021)). A linear actuator (PQ-12) was used to move five prosthetic fingers with a maximum pulling capacity of 50 N for each. However, a linear actuator requires a large power supply (stall current: 550 mA × 5 linear actuators); therefore, it consumes a substantial amount of current. In this study, the linear actuator only performed run and stop movements for two ranges of maximum (20 mm) and minimum (0 mm) strokes. In further research, prosthetic hand movements could be developed into movements that are proportional to the EMG amplitude level. Therefore, the prosthetic hand could imitate the natural movements of human hand.

## 5. Conclusions

This study developed and evaluated an embedded time-domain feature and machine learning on a system on chip (SoC) based on a Raspberry platform using a multi-thread algorithm to control a prosthetic hand device. Our findings showed that MAV and decision tree (DT) resulted in a higher accuracy (98.41%) than other features and machine learning methods. Overall, the training and testing process used in scheme 1 produced a higher accuracy than scheme 2. The processing time required by the system from data acquisition to classification was on average ~1 ms (for NB, *k*-NN, and DT) whereas the average execution time required by machine learning SVM was longer (1.7989 ms). Further developments from this research are to increase the number of machine learning methods and features used to obtain better results.

## Figures and Tables

**Table 1:** Comparison of accuracy between the proposed study and other related studies.

System Parameter	Ref. [19]	Ref. [20]	Ref. [21]	Ref. [18]	Proposed Study
Implementation	Computer	Embedded	Computer	Embedded	Embedded
Training stage	Offline	Offline	Offline	Online	Offline
Testing stage	Online	Online	Online	Online	Online
Machine learning	SVM	SVM	ANN	LDA	DT
Number of motions	9	7	6	6	4
Number of channels	4	8	8	8	2
Accuracy	90%	90%	93.20%	94.14%	**98.13%**
